# Primary Cutaneous Anaplastic Large Cell Lymphoma: A Review of Diagnosis and Treatment for the General Oncologist

**DOI:** 10.3390/cancers18101560

**Published:** 2026-05-12

**Authors:** Jackson T. Bowers, Jasmine Zain

**Affiliations:** 1 Department of Medicine, University of California San Francisco, San Francisco, CA 94143, USA; 2 Lymphoma Service, Department of Medicine, Memorial Sloan Kettering Cancer Center, New York, NY 10026, USA

**Keywords:** primary cutaneous anaplastic large cell lymphoma, CD30, cutaneous T-cell lymphomas, brentuximab vedotin

## Abstract

Primary cutaneous anaplastic large cell lymphoma (pcALCL) is a type of cutaneous T-cell lymphoma (CTCL) classified as a CD30+ lymphoproliferative disorder along with lymphomatoid papulosis. Although it is the second most common subtype of CTCL after mycosis fungoides/Sézary syndrome, it remains rare, with an incidence of fewer than 0.5 cases per million person-years. Despite its histologic similarity to systemic anaplastic large cell lymphoma, pcALCL follows a largely indolent course with excellent outcomes, with disease-specific survival rates of 86–95% in contemporary series. This narrative review summarizes the clinical presentation, diagnostic evaluation, and management of pcALCL based on a semi-structured literature search, with emphasis on prospective studies and clinically relevant retrospective data. Diagnosis remains challenging due to overlap with other CD30-positive lymphoproliferative disorders and reactive conditions, requiring careful clinicopathologic correlation and exclusion of systemic disease. While regional lymph node involvement may be present, available evidence suggests it does not significantly impact prognosis, highlighting the importance of avoiding overtreatment. Management strategies are guided by disease extent, with strong evidence supporting skin-directed therapies, particularly radiation, for localized disease. For multifocal or relapsed disease, brentuximab vedotin demonstrates the most robust prospective data and has reshaped the treatment landscape; however, alternative systemic therapies, including methotrexate and retinoids, remain relevant in selected patients. Overall, current management is supported largely by non-randomized data, and key gaps remain in risk stratification, optimal sequencing of therapies, and management of uncommon aggressive variants.

## Simple Summary

Primary cutaneous anaplastic large cell lymphoma (pcALCL) is a cutaneous T cell lymphoma defined by expression of CD30. Though it is considered the cutaneous counterpart of systemic ALCL, it has a more indolent course characterized by excellent disease-specific survival and does not require treatment with multiagent chemotherapy. Diagnosis is challenging and requires exclusion of systemic lymphoma. Management for solitary disease consists of localized therapies, particularly radiation. For more widespread disease, the anti-CD30 antibody–drug conjugate brentuximab vedotin has recently been approved, and a number of novel therapies are under development. Though it is rare, oncologists should familiarize themselves with the work up and treatment of pcALCL to avoid misclassification as systemic ALCL and avoid overtreatment.

## 1. Introduction

Primary cutaneous anaplastic large cell lymphoma (pcALCL) is a rare cutaneous T cell lymphoma (CTCL) that is classified by the World Health Organization (WHO) as a CD30+ T cell lymphoproliferative disorder (LPD). CD30+ LPDs are the second most common type of CTCL and encompass pcALCL, lymphomatoid papulosis (LyP) and borderline lesions (cases in which pathology and clinical presentation do not align) [1,2,3]. CD30+ LPDs are defined by expression of the CD30 antigen, a cell surface cytokine receptor and transmembrane glycoprotein of the tumor necrosis factor receptor superfamily member 8 (TNFRSF8) gene, previously known as Ki-1 antigen [4].

Diagnosis of pcALCL can be challenging, requiring differentiation from other CD30+ LPDs and excluding systemic lymphoma. Despite being the cutaneous counterpart of systemic ALCL (sALCL), pcALCL has a more indolent course and better outcomes. While systemic ALCL requires treatment with multiagent CHOP-like chemotherapy, pcALCL is typically managed with localized therapy or single agent systemic treatments for more widespread disease. Awareness among oncologists is important to avoid overtreatment for this population. Moreover, the recent introduction of anti-CD30 antibody–drug conjugate brentuximab vedotin has changed treatment paradigms for pcALCL.

This review addresses these needs by summarizing the clinical presentation and staging, pathologic and molecular features, and treatment approach for pcALCL, incorporating the latest studies. A semi-structured literature search was performed using PubMed/MEDLINE, Embase, and ClinicalTrials.gov from database inception through April 2026. Articles were included based on relevance to the topic, with emphasis on prospective clinical trials, key retrospective studies, and seminal translational work. Given the narrative nature of the review, no formal assessment of study quality or risk of bias was performed.

## 2. Epidemiology

CD30+ LPDs, while rare, are the second most common CTCL after mycosis fungoides (MF), representing ~25% of cases. Of the CD30+ LPDs, pcALCL is slightly less common than LyP, accounting for 8% of all CTCLs [5]. Estimates of incidence are limited, but one analysis of the U.S. Surveillance, Epidemiology, and End Results (SEER) database reported an incidence of 0.12 cases per million person-years [6]. European studies have reported an overall incidence of CTCL ranging from 2.9 to 3.9 cases per million person-years, suggesting that, as a subset of CTCL, the incidence of pcALCL is approximately 0.23–0.31 cases per million person-years [7].

pcALCL has a slight male predominance, and most patients present in middle or older age, with a median age of presentation of 61. In contrast, LyP typically presents in the third or fourth decade of life with a median age of diagnosis of 42 [8]. Unlike sALCL, the ALK+ variant of which typically affects children and young adults, pcALCL is rare in children. However, pediatric cases have been described in case series: a large ALCL trial identified 33 patients with primary cutaneous disease, five of whom were ALK-positive (ALK+) [9]. ALK positivity may be more frequent in the pediatric subset, but pcALCL in adults remains characteristically ALK-negative (ALK-), and ALK-positivity in this group should raise suspicion for systemic disease.

Risk factors for pcALCL are not well described. Like other CTCLs, pcALCL may be associated with immunosuppression and has been reported as a complication of solid organ transplant and immunosuppressive medications including tumor necrosis factor-alpha (TNFa) inhibitors [10,11,12]. pcALCL has been reported as a secondary malignancy developing after treatment with CAR-T cell therapy for B-cell lymphoma, though this risk appears to be low [13]. Patients with HIV/AIDS, in addition to being at risk for B-cell lymphomas, are at higher risk for CTCLs including pcALCL [14,15]. In one case series of 25 patients with HIV/AIDS and cutaneous lymphomas, 15 of these were CD30+ T-cell lymphomas [16].

## 3. Clinical Presentation and Differential Diagnosis

PcALCL typically presents as a solitary erythematous skin papule or nodule or grouped eruptions of nodules that can rapidly enlarge and become ulcerated. One study found the most involved areas to include the lower extremities (26%), head and neck (22%), upper extremities (19%), and trunk (18%) [6]. Approximately 20–30% of patients have multifocal involvement with multiple skin areas involved [17,18]. LyP tends to present with smaller more numerous papules compared to pcALCL, but otherwise the lesions can be clinically and histologically similar (Table 1). Importantly, LyP is defined by its self-resolving nature: lesions typically subside on their own after a period of 3–12 weeks, while pcALCL lesions persist without treatment [5,19]. It is not always possible to distinguish LyP and pcALCL based on histologic criteria. Thus, an initial observation period of several weeks may be appropriate to aid in diagnosis and determine whether further treatments are needed.

Diagnosis typically occurs in the dermatologist’s office with a punch or excisional biopsy of lesions. Given its rarity, pcALCL is frequently incorrectly diagnosed and may be initially mistaken for arthropod bites or benign inflammatory skin nodules [20]. Pathologically, the differential diagnosis for pcALCL includes skin involvement of sALCL, other CTCLs including the tumor stage of MF/Sezary syndrome (SS), classical Hodgkin lymphoma (HL) with skin involvement, or non-malignant conditions including lymphomatoid drug reactions and viral infections [21,22]. Lymphomatoid drug reactions may occur after exposure to numerous common medications including statins, cephalosporins, amlodipine, carbamazepine, and sertraline among others. Biopsies of these eruptions may contain CD30+ T cells, which can lead to a false diagnosis of pcALCL; so, a careful medication history should be taken to exclude drug reaction [23,24].

### 3.1. Regional Lymph Node Involvement

Approximately 5–10% of patients with pcALCL may have regional lymph node involvement on diagnosis. Counterintuitively, locoregional lymph node involvement does not appear to be associated with a significantly worse prognosis. In one of the largest cohorts of pcALCL patients studied to date, patients with regional node involvement (defined as involvement of a single nodal station) had a 5-year disease-specific survival of 91% vs. 96% for skin-limited disease [17]. The biologic basis for this observation remains unclear; it is presumed that the indolent biology of pcALCL is such that regional lymph node involvement represents an extension of localized disease rather than the initial phase of widespread dissemination [17]. Regional lymph nodes should be biopsied to rule out other systemic lymphomas, but if the staging workup does not show distant or widespread nodal involvement, these patients do not require treatment with chemotherapy.

### 3.2. Extensive Limb Disease

A variant of pcALCL with extensive limb disease (ELD) has been described and has a more aggressive course presenting with multiple skin tumors in one limb. One study that included four ELD patients showed a 2-year disease-specific survival of 50% compared to 93% for those with typical pcALCL. While the small number of patients limits conclusions, these data suggest that ELD may be associated with inferior prognosis. This study found that ELD was associated with upregulation of the T-cell activation genes STAT5A and IL2R, which may explain its more aggressive course, and downregulation in RXRA pathways, which may confer resistance to topical retinoids [25].

### 3.3. Clinical Course and Prognosis

Overall, pcALCL generally has an indolent course and excellent clinical outcomes. Cutaneous relapse is common with approximately 40–50% of patients experiencing relapse which may require further treatment [4,18,19]. However, survival remains excellent: in recent case series, reported 5-year disease-specific survival rates range from 86 to 95% and 5-year overall survival rates range from 76 to 96% (Table 2) [6,17,18,26,27,28]. This lies in contrast to systemic ALCL, which has a worse prognosis: the ALK+ variant of sALCL has 5-year OS rates of 70–90%, while the ALK- variant has a 5-year OS of only 40–60% [29].

Several clinical factors have been described that are associated with inferior outcomes in pcALCL. Fernández-de-Misa and colleagues showed that progression to nodal involvement during follow up was associated with inferior disease-specific survival with an HR 8.74 (95% CI 1.65–46.29) [18]. Similarly, Liu et al. showed that nodal progression was associated with worse PFS [26]. The extent of skin involvement has also been associated with inferior outcomes, with T2-T3 lesions having slightly worse PFS and DS in addition to ELD as described above [18,26]. Lastly, early cutaneous relapse was associated with worse prognosis in one study [18]. Beyond these somewhat intuitive adverse prognostic factors, demographic or molecular subgroups with worse outcomes have not been well defined.

While uncommon, there is some risk for progression of pcALCL to systemic lymphoma: reported estimates of progression range from 10% for all extracutaneous relapse (including regional lymph nodes) to 14% progression to sALCL [17,28]. In a recent single-center cohort study of 109 patients with CD30+ LPDs including 44 with pcALCL, seven patients went on to develop systemic lymphoma (sALCL in five and HL in two) with a median time from LPD to systemic lymphoma diagnosis of 9.8 years [30]. This underscores the need for biopsy of any suspicious lymph nodes at relapse, even years into remission.

## 4. Pathologic and Molecular Features

### 4.1. Histology

Histologic examination of pcALCL lesions shows a diffuse dermal infiltrate of cohesive sheets of large cells with an anaplastic, pleomorphic, or immunoblastic morphology. Epidermotropism or migration of lymphocytes to the skin surface is absent which can help distinguish it from MF [4]. The “hallmark” cells that define systemic ALCL are also seen in pcALCL. These are large cells with eccentric horseshoe or kidney shaped nuclei and prominent Golgi apparatus [21,31]. Several other histologic variants have been described including an angioinvasive variant with a cytotoxic (CD8-positive) phenotype, which may be mistaken for more aggressive systemic lymphomas, and a pyodermic/neutrophil rich variant that contains a robust inflammatory infiltrate and may be seen in immunocompromised individuals [32,33]. There is no clear prognostic difference based on histologic appearance or subtype [4].

### 4.2. Immunohistochemistry

By definition, CD30 should be positive in more than 75% of pcALCL cells based on the WHO/EORTC criteria for CD30+ LPD [5]. CD30 is a cell surface cytokine receptor and transmembrane glycoprotein of the tumor necrosis factor receptor superfamily member 8 (TNFRSF8), previously known as Ki-1 antigen, that is also expressed on Reed–Sternberg cells in HL, in sALCL, and can also be expressed in other cutaneous lymphomas including MF and in some inflammatory states [34]. Otherwise, pcALCL shares the same immunophenotype as ALK- sALCL, with the expression of CD45, CD43, MUM1/IRF4, and CD4 and with variable expression of CD2, CD5, CD7, and CD45RO [21]. Cases of pcALCL are almost always negative for ALK rearrangement, which occurs in 45–60% of systemic ALCL and is most often associated with t(2;5) chromosomal translocation resulting in the NPM:ALK fusion protein [29,34]. Unlike sALCL, pcALCL is typically positive for cutaneous lymphocyte antigen (CLA) but negative for epithelial membrane antigen (EMA) [4,21]. Staining for Epstein–Barr virus (EBV) is characteristically negative in pcALCL [21].

### 4.3. Molecular Pathology

The molecular pathogenesis of pcALCL remains unclear. In a recent large gene expression profiling study of systemic and cutaneous ALCL, no consistent genetic signature was identified for pcALCL, suggesting molecular heterogeneity [35]. The majority of cases are monoclonal in origin, with 90% of cases showing clonal rearrangement of T-cell receptor genes [36]. *DUSP22* rearrangement at 6p25.3, found in ~30% of ALK- sALCL, has also been well-described in pcALCL [35,37,38,39]. Recent case series have found rates of *DUSP22* rearrangement ranging from 40 to 60% in pcALCL [40,41]. In systemic ALK- ALCL, *DUSP22* rearrangement is thought to confer a prognostic benefit, but in pcALCL, it has not been associated with differential outcomes [37,40]. The role of *DUSP22* in pcALCL oncogenesis has not been fully elucidated, but in systemic ALCL, it is closely associated with the downregulation of *JAK/STAT* signaling pathways, particularly loss of *STAT3* [35]. *TP63* translocation which is rare in sALCL but confers a worse prognosis is exceptionally rare in pcALCL [42].

Early whole-genome profiling studies in pcALCL identified chromosomal instability in 40% of cases, with common copy number alterations including gains of 1/1p and 5 in half of cases [43]. More recent studies have identified alterations in *PI3K/AKT*, *MAPK*, and G-protein signaling pathways, with recurrent mutations in *LRP1B, PDPK1*, and *PIK3R1* and deletions of *PRDM1* and *TNFRSF14* [44]. Further studies are needed to determine the prognostic and therapeutic significance of these molecular findings.

## 5. Work up and Staging

A diagnostic skin biopsy (punch, incisional or excisional) of a representative lesion is required and should be sent for histopathologic evaluation, immunophenotyping, and gene rearrangement studies for full evaluation. Care must be taken to ensure adequate tissue for evaluation. Shave biopsies and fine needle aspirates are considered suboptimal for a proper diagnostic evaluation. The IHC panel may include CD3, CD4, CD8, CD20, CD30, CD56, and ALK. The expanded IHC panel may include CD2, CD5, CD7, CD25, TIA1, granzyme B, perforin, IRF4/MUM1, EMA, and TCRB1. FISH studies to detect ALK and *DUSP22* gene rearrangements may be useful in certain circumstances. ALK rearrangement positivity should call the diagnosis of pcALCL into question and prompt a search for evidence of systemic lymphoma [4].

**Figure 1:**
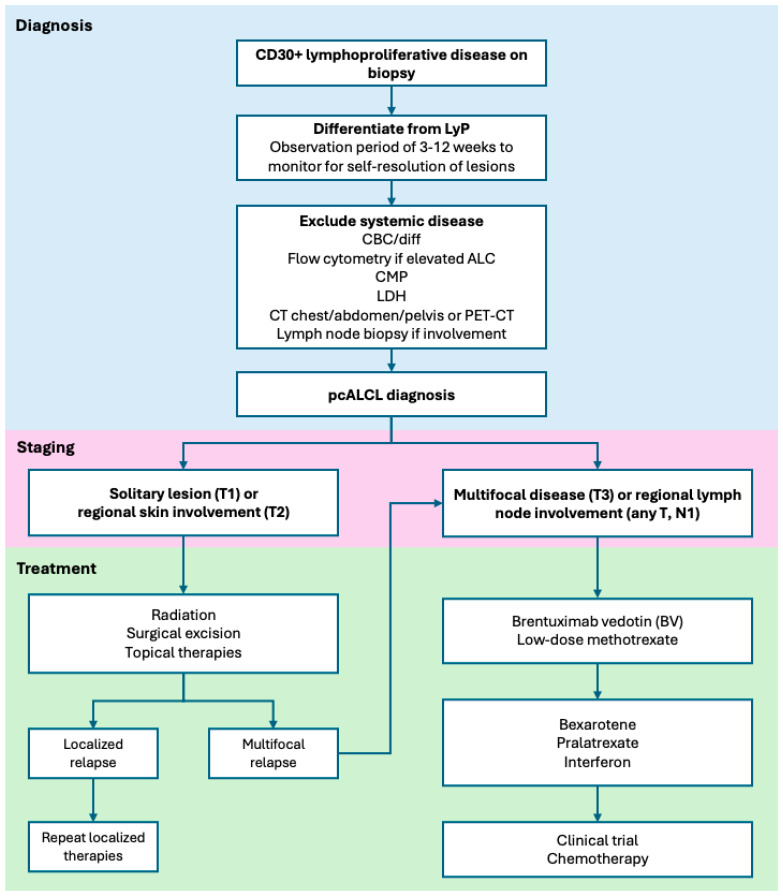
Proposed algorithm for diagnosis, staging and treatment of primary cutaneous ALCL. Key points of pcALCL management including differentiation from LyP, exclusion of systemic disease with staging workup including TNM classification. Localized disease can be managed with radiation therapy +/− surgical excision, while brentuximab vedotin (BV) or methotrexate are first line options for multifocal disease.

The diagnostic workup for pcALCL is shown in Figure 1. When biopsy and clinical context are consistent with a CD30+ LPD, an initial observation period may be appropriate to monitor for self-resolution of symptoms, which suggests LyP rather than pcALCL. If lesions persist, a full clinical evaluation is needed to rule out systemic ALCL including a complete blood count, complete metabolic panel and LDH. If lymphocytosis is present, flow cytometry on peripheral blood should be performed to rule out blood involvement by MF/SS. A CT scan of the chest, abdomen and pelvis with contrast or integrated whole body FDG-PET/CT (arms/legs) should be undertaken for a full body and nodal assessment of disease. Biopsy of enlarged lymph nodes or suspected extracutaneous sites is required to rule out systemic lymphomas. Bone marrow biopsy is indicated if unexplained cytopenias are present but not otherwise required for the diagnostic work up of cutaneous lymphomas [22,45,46]. HIV serology should be obtained, and HTLV1 serology can help establish a diagnosis of HTLV1-associated T-cell lymphoma, especially if there is clinical suspicion or strong positivity for CD25 [16].

Staging for pcALCL is performed according to the International Society of Cutaneous Lymphomas (ISCL)/EORTC guidelines with T1 disease describing a solitary lesion, T2 describing regional skin involvement (multiple lesions limited to one body region or two contiguous body regions) and T3 describing generalized skin involvement defined as involving two noncontiguous or three or more body regions [47] (Figure 1). As discussed, N1 disease can be seen in pcALCL, but N2-N3 disease suggests systemic lymphoma, as does any evidence of visceral involvement (M1).

## 6. Treatment

PcALCL has a unique biology requiring less intensive treatment options than systemic lymphomas. Because it is rare, dedicated randomized controlled trial data are limited; rather, most evidence for treatments in pcALCL comes from cohort studies or case series of multiple types of cutaneous lymphomas. Prior to the landmark analysis by Bekkenk et al. in 2000 [17], it was common for pcALCL to be treated similarly to sALCL with multiagent chemotherapy (CHOP) in combination with radiation [17,28]. That paper along with updated expert recommendations and novel therapies like brentuximab vedotin have shifted management away from chemotherapy [46]. Today, treatment approach consists of localized therapies for solitary lesions and consideration of systemic therapy (but rarely chemotherapy) for multifocal or relapsed disease (Figure 1).

### 6.1. Skin-Directed Therapies

#### 6.1.1. Radiation Therapy and Surgical Excision

Radiation therapy, typically involved-site radiation therapy (ISRT), is recommended as the first line therapy for single or grouped lesions, either alone or in combination with surgical excision [22,46]. Historically, higher radiation doses (30–40 Gy) were used, but lower doses achieve excellent outcomes, and a total dose of 24 Gy is now typically recommended [22,48,49]. Smith et al. found that even doses of <20 Gy were associated with three-year freedom from local relapse of 100% [49]. RT can be an appropriate treatment even for selected patients with multifocal disease: the Dutch Cutaneous Lymphoma registry found a 100% complete response (CR) rate for 21 patients with multifocal disease treated with RT, though none of these patients had more than five lesions [50]. At cutaneous relapse, patients can be re-treated with radiation, and palliative doses as low as 2–4 Gy are recommended [51]. Radiation is typically very well tolerated with mild localized side effects including itching and erythema [52].

Surgical excision may be considered as an alternative or in combination with radiation as upfront therapy. Limited data are available about the effectiveness of surgical excision, but a pooled analysis suggests cutaneous relapse rates of ~40%, on par with general relapse rates for the disease [46]. It is included in the treatment guidelines as an alternative for solitary or grouped lesions [22].

#### 6.1.2. Topical Therapies

Several topical therapies have been reported to be effective in pcALCL, including topical steroids, imiquimod, topical nitrogen mustard, topical bexarotene (a vitamin A derivative), and laser treatments [46]. These therapies can be used alone or in conjunction with radiation therapy. While definitive evidence for these therapies is lacking, they may be considered for localized disease [22,46]. Side effects of topical therapies commonly include burning, localized skin irritation and/or contact dermatitis. A dermatologist should be involved in co-managing patients with pcALCL given their expertise in these therapies.

### 6.2. Systemic Therapies

Systemic therapy is reserved for patients with extensive multifocal involvement or those whose disease is not adequately controlled by local therapies.

#### 6.2.1. Brentuximab Vedotin

Brentuximab vedotin (BV) has recently become a recommended first line treatment for multifocal disease or regional lymph node involvement. BV is a humanized CD30 antibody conjugated to monomethyl auristatin E (MMAE), an anti-tubulin agent that is delivered as a cytotoxic payload to CD30+ cells. BV is approved for the treatment of HL, sALCL, and other CD30-expressing lymphomas.

BV was first studied in pcALCL in a phase II clinical trial of 48 patients with CD30+ CTCL, including 28 patients with CD30+ MF. The overall response rate (ORR) was 73% with 25% CR. Patients with pcALCL or LyP had an ORR of 100% vs. 54% for those with MF [53]. Building off this study, the ALCANZA trial was a randomized phase 3 study of BV vs. physician’s choice (methotrexate or bexarotene) in 128 patients with CD30-expressing cutaneous lymphomas who had received previous treatment, again including CD30+ MF (*n* = 97) in addition to pcALCL (*n* = 31 [24%]). In the overall cohort including MF patients, those treated with BV had superior outcomes compared to physician’s choice including a durable 4-month ORR (ORR, 54.7% vs. 12.5%), CR (17.2% vs. 1.6%), and median PFS (mPFS, 16.7 months vs. 3.5 months). These outcomes held when limited to the 31 patients with pcALCL: ORR4 was 75% with BV vs. 20% with physician’s choice and CR rate 31% vs. 7% with mPFS 27.5 months vs. 5.3 months [54]. A recent real-world cohort study in the US showed similarly improved outcomes with BV compared to other standard therapies in the second line setting [55].

BV is typically administered at a dose of 1.8 mg/kg once every 3 weeks, for up to 16 3-week cycles [54]. The most significant toxicity from BV is peripheral neuropathy, occurring in 67% of patients in ALCANZA (grade 2 in 21 patients, grade 3 in 6 patients) [54]. At final analysis, 86% of patients with neuropathy had complete resolution or improvement to grade 1–2, with 18 patients having ongoing grade 1–2 neuropathy [56]. Other grade 3–4 events include neutropenia, nausea, and fatigue [54] Very rarely, BV has been associated with reactivation of the John Cunningham (JC) virus leading to progressive multifocal leukoencephalopathy (PML), an often fatal central nervous system infection [57]. Early cases of PML led the FDA to place a boxed warning on the label of BV for JC virus reactivation.

The ALCANZA trial showed that BV treatment resulted in improved clinically meaningful durable responses and time to next treatment, supporting a role as a treatment for relapsed disease or cases of multifocal or nodal involvement. However, considerations of availability, route of administration, and toxicity profile may lead clinicians to choose alternative options (e.g., methotrexate) initially for specific patients. The cost of BV may also be prohibitive, though one Canadian analysis found that BV was cost-effective compared to methotrexate or bexarotene in a model of a population of patients with MF and pcALCL [58].

#### 6.2.2. Methotrexate

Methotrexate inhibits dihydrofolate reductase and belongs to the antimetabolite class of cytotoxic chemotherapies with a wide range of clinical oncologic and non-oncologic indications. Low-dose methotrexate (10–50 mg weekly) is effective in pcALCL and recommended in cases of multifocal involvement that either do not respond to BV or in which BV is contraindicated [22]. Durable ORR was low for the methotrexate group in ALCANZA (7.7%), but other studies have shown response rates up to 57% with CR rates of 43% [50,54]. It can be combined with skin-directed therapies. The main side effects of low-dose methotrexate are mucositis, myelosuppression, and gastrointestinal symptoms. Liver and pulmonary toxicity can also occur, warranting close monitoring. Since liver fibrosis has been reported with long-term methotrexate use, it is reasonable to implement a treatment-free interval after a maximum of three years of maintenance therapy. Concomitant use of folic acid may reduce the risk of mucosal toxicity [59,60,61].

#### 6.2.3. Bexarotene

Bexarotene is a systemic retinoid that can be used for multifocal disease not responsive to other therapies. It selectively binds retinoid receptors in the nucleus that dimerize and then bind to specific DNA sequences called retinoic acid response elements (RAREs) involved in the regulation of cell growth, differentiation, and inflammation. Oral bexarotene is typically used at a dose of 300 mg/m^2^ daily for cutaneous lymphomas. Limited data from case reports are available to support its efficacy [62,63]. In the ALCANZA study, the bexarotene group had a 4-month durable ORR of 12.5%, inferior to BV [54]. Nonetheless, it may be an option for multifocal relapsed disease. The main side effects of bexarotene include hyperlipidemia and hypothyroidism, which are common and may necessitate dose reduction [64].

#### 6.2.4. Pralatrexate

Pralatrexate is a dihydrofolate reductase inhibitor like methotrexate but is designed to have high affinity to its receptor and be more efficiently internalized into cancer cells. It is approved to treat relapsed/refractory peripheral T-cell lymphomas and also has shown efficacy in CTCL. In a phase II study of 54 patients with CTCL including one patient with pcALCL, the ORR was 45%, and the patient with pcALCL had a CR [65]. A subsequent phase I/II combination study of pralatrexate plus bexarotene in 34 patients with relapsed/refractory CTCL achieved a 60% ORR [66]. Pralatrexate is administered intravenously at a dose of 15 mg/m^2^ weekly for 3 weeks of each 4-week cycle. The main side effects are thrombocytopenia and mucositis, with grade 3 mucositis occurring in 17% [65]. Pralatrexate may be an option for extensive disease in cases refractory to BV or methotrexate; notably, responses have been observed even in patients who progressed on methotrexate despite their similar mechanism of action [65].

#### 6.2.5. Interferons

Alpha interferon is an immunomodulatory agent that is effective in treating other CTCLs including MF/SS. While evidence specifically for the treatment of pcALCL is lacking, several case series have shown efficacy for interferon in LyP [67,68,69], and it is recommended in guidelines as an treatment option for progressive multifocal disease [22]. One case report described a patient with pcALCL achieving CR after treatment with a combination interferon alpha with bexarotene [70]. The main adverse effects of interferon include leukopenia, thrombocytopenia, transaminitis, fatigue, and weight loss [68].

#### 6.2.6. Chemotherapy

Use of multiagent chemotherapy is not indicated in cases of pcALCL, unless there is evidence of systemic disease, in which case guidelines for systemic T cell lymphoma should be followed. In some refractory cases of aggressive presentations, single agent chemotherapy may be considered with gemcitabine, pegylated doxorubicin or etoposide; patients with pcALCL have been included in trials for each of these agents [71,72,73]. Pegylated doxorubicin has been shown to be very effective as a single agent presumably due to the increased efficacy of liposomal doxorubicin in the skin [72].

#### 6.2.7. Role of Stem Cell Transplantation

Stem cell transplant is indicated only in cases of pcALCL with highly refractory disease or progression to systemic lymphoma. These patients are treated according to the guidelines for systemic T cell lymphomas and may require transplant for long-term disease control. Allogeneic stem cell transplant (alloSCT) has been studied in advanced CTCL (MF/SS) in the CUTALLO trial, demonstrating an mPFS of 9.0 months compared to 3.0 months without transplant, but no patients with pcALCL were included in this trial, and the overall survival benefit was not statistically significant [74]. In patients who have transformation to sALCL, allogeneic transplant appears to be an effective option for long-term disease control for a subset of patients: the largest retrospective cohort study to date of 182 patients with sALCL who underwent alloSCT reported a 5-year PFS and OS of 41% and 56%, respectively [75].

#### 6.2.8. Novel Therapies

Several new agents have recently been approved for CTCLs and may hold promise for pcALCL. These include the anti-CCR antibody mogamulizumab and denileukin diftitox-cxdl, a recombinant fusion protein of diphtheria toxin and human interleukin-2 that was recently re-approved for relapsed/refractory CTCL [76,77]. Immune checkpoint inhibitors appear to be active in CTCL: a phase II trial of pembrolizumab demonstrated an ORR of 38% in a heavily pretreated population, and a more recent phase I/II trial of durvalumab in combination with lenalidomide showed an acceptable safety profile and ORR of 58% with a median duration of response of 25.5 months [78,79]. Agents with novel mechanisms of action are also being investigated including anti-CD70 antibodies, the anti-KIR3DL2 antibody lacutamab, and JAK and PI3 kinase inhibitors [80,81].

Novel CD30-targeting agents are also under development. Anti-CD30 CAR-Ts have been trialed in patients with HL and CD30+ T-cell lymphomas, though the efficacy has been mixed, particularly for patients with ALCL in these studies [82,83,84,85]. Similarly, a phase I study of allogeneic NK cells complexed with a bispecific CD30/CD16 antibody showed a high CR rate in patients with HL but a comparatively worse response rate in CD30+ NHL [86]. Further studies are needed to understand why ALCL may be more resistant to cellular therapies. Other CD30-targeting therapies being trialed including bispecific antibodies and next-generation ADCs [87,88,89].

## 7. Conclusions

Primary cutaneous anaplastic large cell lymphoma is considered the cutaneous counterpart of systemic ALCL. However, the two entities have distinct clinical features, treatment and prognosis. pcALCL can be difficult to diagnose with clinicopathologic similarity to LyP and other CD30-expressing CTCLs, and care must be taken to exclude systemic disease. But in the absence of systemic involvement, the prognosis is excellent, and treatment typically involves radiation therapy or targeted agents like brentuximab vedotin for multifocal disease, sparing patients from multiagent chemotherapy regimens used to treat systemic ALCL.

As the present study is a non-systematic narrative review, it may be limited by selection bias. Future studies should focus on understanding the biology and clinical behavior of rare phenotypes of pcALCL including ELD and regional lymph node involvement to determine whether these require alternative treatments. Further elucidation of the molecular drivers of pcALCL is needed to understand what targeted therapies may be most active in this disease. Lastly, better characterization of risk factors for transformation to systemic lymphoma could help identify this minority of patients earlier.

## Figures and Tables

**Table 1:** Comparison of CD30+ lymphoproliferative disorders and systemic ALCL.

	Primary Cutaneous ALCL (pcALCL)	Lymphomatoid Papulosis (LyP)	Systemic ALCL (sALCL)
Clinical presentation	Solitary or grouped erythematous papules or nodules	Recurrent self-healing papules or nodules; often multiple, waxing and waning, may leave scars	Rapidly progressive lymphadenopathy, B symptoms; may involve extranodal sites (skin, bone, lung)
Histologic features	Dermal infiltrate of large anaplastic CD30^+^ cells; cohesive sheets; minimal epidermotropism	CD30^+^ atypical lymphocytes in a mixed inflammatory background; variable patterns (types A–E)	Sheets of large anaplastic CD30^+^ cells; hallmark cells; sinusoidal growth pattern in lymph nodes
Molecular features	Characteristically ALK^−^; clonal T-cell receptor rearrangement often present; DUSP22 rearrangements in subset	Usually ALK^−^; clonal T-cell receptor rearrangement may be present	ALK^+^ (e.g., NPM1-ALK fusion) or ALK^−^; ALK^−^ often harbors DUSP22 rearrangement and rarely TP63 rearrangements
Course and Prognosis	Indolent; excellent prognosis (>90% 5-year survival)	Chronic, relapsing but self-resolving; excellent prognosis; small risk of associated lymphomas (e.g., MF, Hodgkin lymphoma, ALCL)	ALK^+^: more favorable prognosis, 5-year OS 70–90% ALK^−^: more aggressive course, 5-year OS 40–60%
Treatment	Local therapy (surgical excision or radiation); brentuximab vedotin monotherapy or low-dose methotrexate for multifocal disease	Observation in most cases; low-dose methotrexate or skin-directed therapy if symptomatic	Systemic therapy (e.g., brentuximab vedotin + CHP); targeted therapy based on subtype

**Table 2:** Five-year survival outcomes for primary cutaneous ALCL.

Study	*N*	Study Period	Setting	Median Follow Up Time (mo.)	Five-Year Disease-Specific Survival (DSS)	Five-Year Overall Survival (OS)
Sarfraz et al. 2021 [6] (US SEER database)	501	2015–2016	Multicenter	52	NR	81%
Fernández-de-Misa et al. 2020 [18] (Spanish cutaneous lymphoma group)	108	1986–2017	Multicenter	51	93%	NR
Hapgood et al. 2017 [28] (British Columbia experience)	47	1993–2017	Multicenter	101	86%	75%
Bekkenk et al. 2000 [17] (Dutch cutaneous lymphoma database)	90 *	1983–1998	Multicenter	61	95%	82%
Lee et al. 2016 [27] (Asan medical center)	38	1995–2014	Single center	41	88%	NR
Liu et al. 2003 [26] (Stanford experience)	25	1974–2001	Single center	39	91%	77%

NR = not reported. * Study included 247 patients; data here limited to pcALCL patients (*N* = 79 with skin-limited disease, *N* = 11 with regional node involvement).

## Data Availability

No new data were created or analyzed in this study. Data sharing is not applicable to this article.
